# A survey of experts on personalized medicine landscape in European Union and China

**DOI:** 10.1186/s12913-023-09471-y

**Published:** 2023-05-23

**Authors:** Ilda Hoxhaj, Flavia Beccia, Alisha Morsella, Chiara Cadeddu, Walter Ricciardi, Stefania Boccia

**Affiliations:** 1 Section of Hygiene, Department of Life Sciences and Public Health, Università Cattolica del Sacro Cuore, 00168 Rome, Italy; 2 Interfaculty of Economics and Medicine and Surgery, Università Cattolica del Sacro Cuore, 00168 Rome, Italy; 3 Department of Woman and Child Health and Public Health,, Fondazione Policlinico Universitario A. Gemelli IRCCS, 00168 Rome, Italy

**Keywords:** Personalised Medicine, Survey, Europe, China, Policies

## Abstract

**Introduction:**

Personalized Medicine (PM) is one of the main priorities of the research agenda of the European Commission and the focus of the European Coordination and Support Action titled “Integrating China into the International Consortium for Personalized Medicine” (IC2PerMed). Similar to the European focus, PM is a current priority of the Chinese Government, through dedicated policies and its five-year investment plans. In the context of IC2PerMed, we implemented a survey to understand the state of the art of the implementation of PM related policies in EU and China, and to identify opportunities for future Sino-European collaborations.

**Methods:**

The survey was elaborated by the IC2PerMed consortium and validated by a focus group of experts. The final version, in English and Chinese, was administered online to a pool of accurately selected experts. Participation was anonymous and voluntary. The survey consists of 19 questions in 3 sections: (1) personal information; (2) policy in PM; (3) facilitating and hindering factors for Sino-European collaboration in PM.

**Results:**

Forty-seven experts completed the survey, 27 from Europe and 20 from China. Only four participants were aware of the implementation of PM-related policies in their working country. Expert reported that PM areas with greatest policy impact so far were: Big Data and digital solutions; citizen and patient literacy; and translational research. The main obstacles found were the lack of shared investment strategies and the limited application of scientific developments in clinical practice. Aligning European and Chinese efforts, finding common ground across cultural, social, and language barriers, were considered as actions needed to enhance efforts in applying PM strategies internationally.

**Conclusion:**

To achieve efficiency and sustainability of health systems, it remains crucial to transform PM into an opportunity for all citizens and patients with the commitment of all the stakeholders involved. The results obtained aim to help define common research and development approaches, standards and priorities and increase collaboration at international level, as well as provide key solutions to enable convergence towards a common PM research, innovation, development and implementation approach between Europe and China.

**Supplementary Information:**

The online version contains supplementary material available at 10.1186/s12913-023-09471-y.

## Introduction

Increasing chronic diseases, ageing populations, and enormous technological advancements are driving health systems to seek sustainable and evidence-based interventions capable of reforming the way services are delivered to the population. Within this context, traditionally dominated by the medical standard of ‘one-size fits all’ approaches, many healthcare leaders are trying to understand how personalization can improve quality of care while reducing costs [1]. The rapidly developing science-driven approach of Personalised Medicine (PM) is shifting the focus to tailored treatment, prevention and prediction of diseases [2]. In this regards, decision-makers worldwide are designing policies to implement sustainable solutions to reshape health systems and services and finding mechanisms for more efficient allocation of resources, to reduce the overall costs in health care while creating healthier populations [3].

Since the publication of the PerMed report “Shaping Europe’s Vision for Personalised Medicine”, in 2015, PM has been among the top priorities of the European Union’s (EU) agenda. From then, it started being considered from a broader policy perspective and the European Commission (EC) now supports developments within the International Consortium for Personalised Medicine (ICPerMed), aimed at encouraging joint efforts in PM research and implementation, and funds a large amount of PM related projects and initiatives [4–6].

Likewise, PM is attracting massive interest in the People’s Republic of China, where the term ‘Precision medicine’ is often preferred over ‘Personalised Medicine’. The concept was first proposed in 2006, whereas the first national strategy expert meeting was held in 2015, also formulating a development plan. Starting from the following year, precision medicine was included at the highest governmental guidance level, in the 13th and 14th Five-Year Plans [7, 8] and in the Healthy China 2030 plan [9].

With PM already being placed on the highest political agendas both in the EU and China, great potential lies in the harmonization and standardization of cross-border data sharing and transfer processes, hence collaborations are an important part of the response to the urgent need for international cooperation in PM development, implementation and promotion. In this regard, the EC has funded the project “Integrating China in the International Consortium for Personalised Medicine” (IC2PerMed), which aims to provide key solutions for enabling the convergence towards a common approach towards PM’s uptake [10]. Within this project, we conducted an online expert survey aimed at identifying policies, programs, initiatives and stakeholders, linked to the development and implementation of PM in the EU and China, and ease opportunities for Sino-European research collaborations. This survey, in addition to helping gain a clearer perspective on the meaning and implications of PM in both countries can lead to the identification of common ground or facilitators for future collaborations as well as obstacles to be addressed.

## Methodology

### Survey elaboration

Building on the IC2PerMed project public Deliverable “Scoping paper: Review on health research and innovation priorities in Europe and China” [11], which provided an up-to-date mapping of policies, strategies, programs, and action plans in PM both in EU and China, we elaborated the items to be addressed in the survey. The survey draft, in English, was validated internally by a multidisciplinary focus group of experts, part of the IC2PerMed Consortium. The experts were asked to evaluate whether the questions were clear, and to provide further suggestions. Based on their feedback, the required modifications were implemented, and the final version was approved. Afterwards, the survey was translated in Chinese, and then validated through an internal focus group of Chinese experts, part of the consortium too. Both, the English and Chinese versions were implemented online via the Lime Survey platform.

The final version of the survey consisted of 19 questions, divided in three sections, as follows:

Section 1: Personal details (4 Questions).

Section 2: Policies and agencies in your country (7 Questions).

Section 3: Facilitators and barriers for collaborations between Europe and China in PM (6 Questions).

The survey is available in Supplementary material.

### Expert selection

Experts engaged in PM-related activities were selected through:

- screening of latest reports, scientific publications, conference programs, EU funded projects partners and coordinators.
- IC2PerMed partners’ network.
- a dedicated Open Call for Experts’ Interest’ on the IC2PerMed website (https://www.ic2permed.eu/open-call-for-experts/).

### Survey dissemination

After the selection process reported above, an email invitation was sent to the identified experts asking them to participate in the project activities. To those who agreed, the survey was sent by email on 15 September 2022, specifying that the participation was anonymous, and that the data were treated in accordance with GDPR regulation. After two weeks, all the invitees automatically received a reminder email.

Additionally, the survey was published on the IC2PerMed Project’s website. Data collection took place from 15 September to 01 November 2022.

Data were summarized through a descriptive synthesis, grouped into three categories:

1. Policy planning and development.
2. Research priorities.
3. Sino-European Collaborations.

## Results

The selection process resulted in 55 experts willing to join IC2PerMed project activities. Among them forty-seven (85%) experts completed the survey: 27 from EU and 20 From China. Two of the Chinese respondents were currently working in EU countries, Germany, and Belgium, respectively. The EU respondents conducted their activities in research institutions, patient organizations, hospitals, governments, and private sector organizations, working in the field of cancer and stem cell research, public health, global health, digital health, international research and innovation in healthcare, value-based healthcare, patient preferences and engagement, hospital management, epidemiology, genomics, translational science, and policy development. Chinese experts worked for research institutions in the field of disease control and prevention; neurodegenerative diseases, neuroscience, international cooperation, and policy development.

### Policy planning and development

Only four respondents (3 European; 1 Chinese) were aware of any policies focused on or related to PM in their working country. In general, both European and Chinese experts indicated the following as areas in which PM-related policies had an impact so far:

- bringing innovation to market
- research funding, and
- translating basic to clinical research and beyond.

**Fig. 1:**
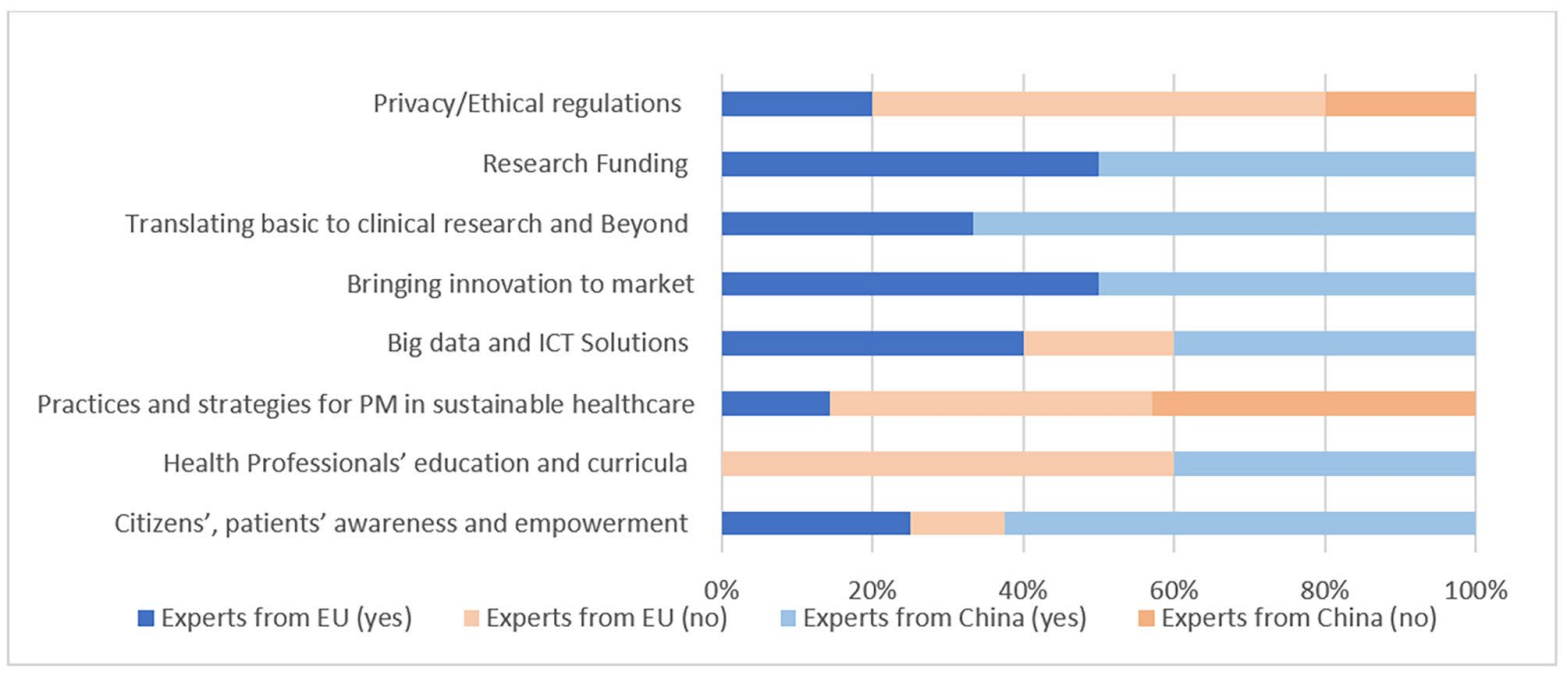
Areas with an impact of policies related to PM according to European and Chinese respondents

Regulations about privacy or ethical aspects, and the development of practices or strategies were considered as less affected areas. According to Chinese experts, policies had influenced the elaboration of healthcare professional curricula, whereas in the EU it was considered as an area without any impact (shown in Fig. 1).

**Fig. 2:**
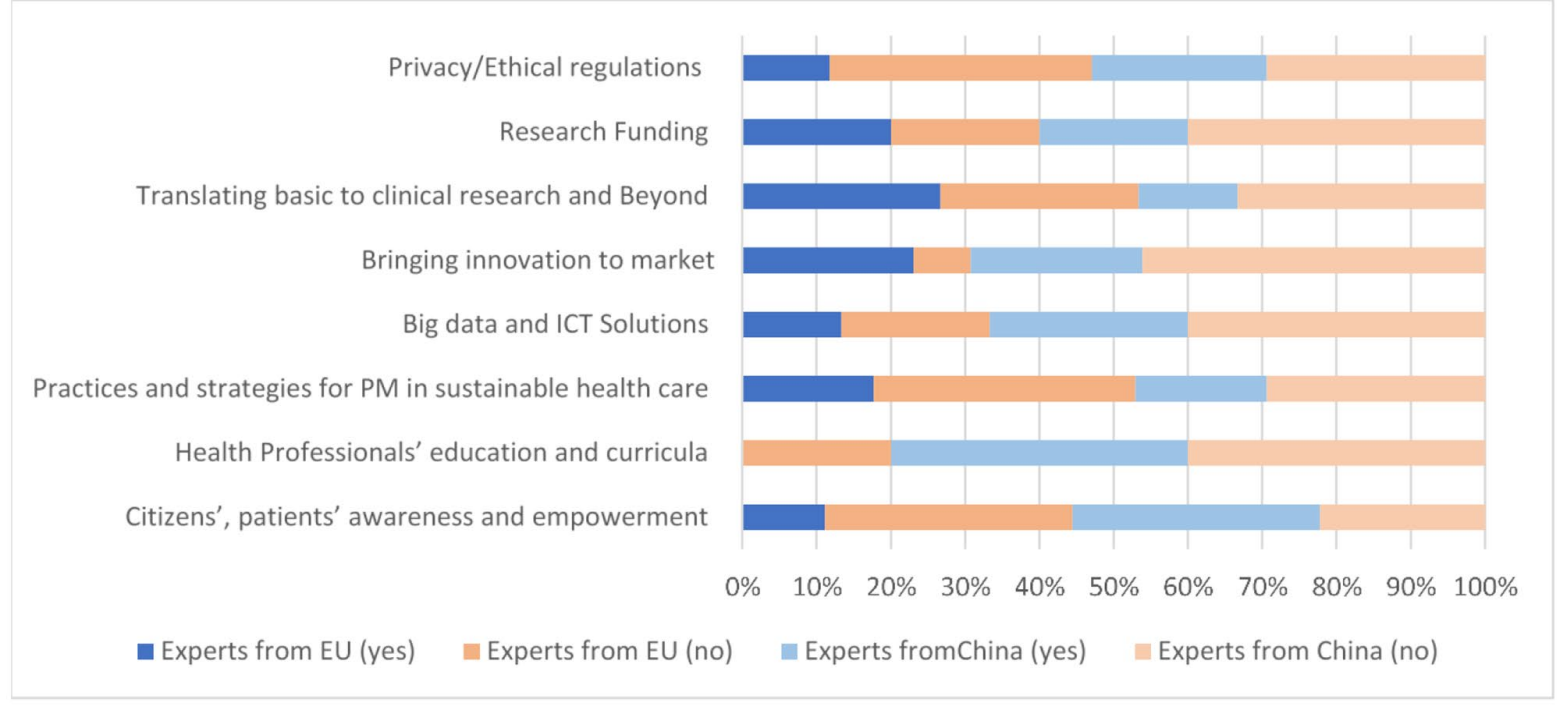
Priority areas to be considered in policy planning in the field of PM according to European and Chinese respondents

Translating basic to clinical research, followed by research funding, has been reported as a priority to be considered in policy planning and implementation in the field of PM. More Chinese experts, compared to European ones, indicated that decision makers should design policies that aim to increase citizens’ and patients’ awareness and empowerment, integrate big data and ICT solutions, and ensure healthcare sustainability. On the other hand, the majority of European experts listed bringing innovation to market as a priority, whereas they did not consider as important the focus at healthcare professionals’ curricula (shown in Fig. 2).

Both European and Chinese, experts agreed lack of research funding strategies and investments in PM as the main obstacle to policy planning, development, and implementation. Additionally, European experts listed the following obstacles:

- integration of research/ experimental setting into routine healthcare;
- predominance of health insurance provider in the market;
- lack of a common understanding of PM;
- non adequate and sufficient education and training of health professionals;
- low citizen’s literacy;

On the other hand, the focus of Chinese participants was mainly at the regulatory aspects, indicating as barriers:

- low awareness on the legal system, and lack of experts with both a medical and a legal background, and related ethical issues;
- communication barriers;
- inadequate assessment of risk-benefit ratios of novel technologies;
- healthcare professionals’ integrity;
- data accessibility, infrastructure, and safety.

### Research priorities

Sixteen participants (30%) indicated the research priorities in PM in their working country. Personalized prevention of chronic diseases, biomarker research, cancer, pharmacogenomics, rare diseases, neurodegenerative diseases, data interoperability and infrastructure were listed as the research priorities by six European experts. According to the ten Chinese participants, the main focus of research has been on next generation sequencing; cell and gene therapy; big data infrastructure and incorporation; new technology; large-scale cohorts and sustainability of health care system.

### Sino-European collaborations

Only three European experts were aware of any collaborations in the field of PM between Europe and China: two of them of IC2PerMed, whereas the other indicated a Sino-German collaboration among Heinrich-Heine University and Beijing Tiantan Hospital, working for *ALDH1A3*, a metabolic instructor of immunity in neuro-oncology.

According to all experts, several contextual aspects, such as social, cultural, economic, ethical, legal should be taken into consideration when establishing EU-China PM-related collaborations. They indicated as the main barriers the differences in the political and legal systems, work ethics, cultural aspects, language and general communication means, physical distance, regulatory instruments, public understanding and acceptance of PM value. Funding opportunities for bilateral activities with common objectives that could allow exchanges of people, knowledge, and ideas; creation of joint institutions, alignment of regulatory and ethical requirements, have been reported as drivers that could facilitate such collaborations. Increased investments in research and innovation in omics technologies, big data, biobanks and ICT solutions, establishment of associations or working expert panels to identify common directions in PM, and in particular of a public reachable media both, in Chinese and English, could contribute to intensifying and promoting collaborations in PM.

## Discussion

Our survey showed that PM is being considered as a relevant issue within the R&I policy and funding landscape in China and the EU. The health research agenda has been reported as disease-focused, that is, national health research priorities have been related to major health problems, such as cancer, chronic diseases or neurodegenerative disease.

Investing in PM research deemed imperative, in both countries, to better understand the molecular mechanisms of diseases, as well as to identify and validate biomarkers. Greater agreement emerged on where policies have the greatest impact, financing and bringing innovation to market being the most relevant. Although China was considered as the second largest spender on R&D globally as of 2020 [12], lack of funding was indicated as the main obstacle for targeted research [13]. A recent study by Wang et al. [14] reported that the Chinese government funding is mainly associated with enhancement of measurable and recognizable scientific output, whereas EU is more likely to fund projects with social impact, that might not directly be transferable to scientific output [15]. The experts considered that partnerships between funding organizations, government, pharmaceutical industry and academic institutions have the potential to facilitate funding programs or activities. It has been widely discussed that the main barriers to PM financing are reimbursement through traditional payment models [15], not specific to PM and a strong link between public and private stakeholders, as well as industry and academia [16]. In China, it is more likely performance based funding for immediate high publication output, whereas EU plays a crucial role in supporting priorities of internal EU market development [14].

The indicated priorities differed across countries, posing challenges to present to the authorities the benefits that PM research represents for the health system. It depends, also, on the level of discordance between research prioritization at national or international agenda. The latter, alongside with lack of harmonization of legal and ethical guidelines are considered by the experts as the main barriers to financing research in PM [17, 18]. Healthcare systems have fragmented legislations and PM policies and programmes vary significantly among countries. Currently, neither China, nor the EC have adopted any decision for cross-border sharing of data, technical resources, or biological material. for optimal cooperation. Data transfer within and outside EU is regulated by the GDPR, whereas within and outside China by the PIPL, with stringent requirements on security controls and data localization [19]. Given that the regulations in place do not consider all the specificities of personalized interventions, it emerges as fundamental need to update or develop the current regulations and harmonize legislation.

European experts focused on translational medicine and innovation, while the Chinese experts pointed out the importance of workforce training and patient involvement. Big data and ICT solutions were a key shared theme, fostered in both countries by significant investments and dedicated policies [19]. The great interest in big data and ICT solutions could be leveraged as a driver for European policies to improve healthcare quality through the rapid and effective implementation of new technologies; similarly, Chinese research institutions could gain new perspectives from the focus on patient and citizen engagement conducted in the EU. Leveraging each other’s experiences and expertise could lay the foundation for new collaborations, creating a virtuous circle of exchange of ideas, knowledge, and best practices [6, 20]. In line with this, our survey emphasized that Sino-European collaborations need to be strengthened, based on mutual partnerships, at both scientific and policy level [21]. To maximize the mutual synergies, existing networks and collaborations should be taken advantage of, given that since the Science and Technology Cooperation Agreement in 1998, EU and China have established a long-term scientific, research and innovation cooperation [22, 23]. In fact, in the EU’s FP7 funding programme (2007–2013), China was the third most important international partner country. In 2015, the Chinese Government and the EU agreed to set up a Co-Funding Mechanism for research and innovation, that could pave the way towards a closer collaboration, further been renewed for the period 2018–2020, to support joint projects between European and Chinese universities, research institutions and companies [23]. Through multilateral activities, EU and China could learn from each other’s experiences and practices on how to tackle certain challenges and share stories of success. However, according to a recent article, the current level of collaborations is suboptimal [24]. Therefore, further actions are needed to facilitate and strengthen such synergies or to launch new ones.

According to our results, policymakers do not fully understand the meaning and exact implications of PM in health systems. The social and ethical consequences of PM implementation have not been fully evaluated, which is why the principles of ELSI, also across-borders, need to be further studied by research and applied by all stakeholders [21]. Within this context, experts raised concerns regarding the cooperation on standardization and research ethics, particularly in China, when refereeing to the adoption of novel technologies. Recently, ethical practices in research have been included in the political agenda in China, where on January 2019, MoST and Ministry of Finance published a joint document urging research institutions and scientists to strengthen ethics regulation and establish regulatory committees [25].

These results should be interpreted in the light of some limitations and strengths. Our sample size might not be representative of all the experts working in PM- related activities, although we conducted an extensive search in identifying them. Therefore, the respondents’ activities were mainly focused in certain domains of PM, thus their interpretation was not general, but domain-driven, being probably subjective. However, to our knowledge, this was the first survey aiming to understand PM scenario, both in China and the EU, in a comprehensive way.

To conclude, specific long term policy measures or strategies focused on PM are required to achieve sustainable development. A continuous and sustainable cooperation between the EU and China, that takes into account the main drivers of change, as well as the relative uncertainties, is fundamental to support networking between European and Chinese policy makers, scientists, programme owners and funders.

## Electronic supplementary material

Below is the link to the electronic supplementary material.

Supplementary Material 1: IC2PerMed survey

## Data Availability

All data generated or analysed during this study are included in this article. Further enquiries can be directed to the corresponding author.
