# Supplementary material for: A survey of experts on personalized medicine landscape in European Union and China
Source: BMC Health Serv Res. 2023 May 23;23:517. doi: 10.1186/s12913-023-09471-y (PMC10204258; doi:10.1186/s12913-023-09471-y)
Supplement: Supplementary file 1 — Supplementary Material 1: IC2PerMed survey [file 12913_2023_9471_MOESM1_ESM.docx]

**Supplementary File 1. IC2PerMed survey**

**SECTION 1: Personal details**(The questions with the symbol # are mandatory)

1. What is your nationality? (Open question) ___________
2. Please indicate the country where you are currently working in # (Open question) __________
3. Please indicate your field(s) of expertise (Open question) __________
4. Which type of organisation or institution are you currently working for?
   1. Government - Research & Innovation
   2. Government - Health
   3. Funding Agency
   4. Innovation/Development Agency
   5. Cluster organisation
   6. Research institution
   7. Hospital
   8. Regulator
   9. Patient organization
   10. Private sector organization (e.g. biotechnology, information technology, pharma, health insurance company)
   11. Other, please specify __________

**SECTION 2: Policies and agencies in the country you are working in**

In this section, you will be asked about your knowledge on policies (including policy measures, programmes, strategies, and action plans), agencies and funders in the field of Personalised Medicine in your respective country.

1. Are you aware of any **policies** focused on or related to Personalised Medicine in the country you are working in?
   - Yes*
   - No
   - I do not know

*If yes, please specify:

- - Name(s)/title(s) __________
  - Source or website, if available __________
  - Any additional information you consider useful __________

*If yes, according to you, in which of the following fields so far have these policies had an impact on Personalised Medicine in the country you are working in (select up to three)?

- - Citizens’, patients’ awareness and empowerment
  - Health Professionals’ education and curricula
  - Practices and strategies for Personalised Medicine in sustainable health care
  - Big data and ICT Solutions
  - Bringing innovation to market
  - Translating basic to clinical research and Beyond
  - Research Funding
  - Privacy/Ethical regulations
  - Other (please specify) ____________
  - I do not know

1. What are the priority areas to be considered in policy planning in the field of Personalised Medicine in the country you are working in?
   - Citizens’ awareness and empowerment
   - Health Professionals’ education and curricula
   - Practices and strategies for Personalised Medicine in sustainable health care
   - Big data and ICT Solutions
   - Bringing innovation to market
   - Translating basic to clinical research and Beyond
   - Research Funding
   - Privacy/Ethical regulations
   - Other (please specify) ___________
2. According to your opinion, what are main obstacles to the planning, development and implementation of policies in the field of Personalised Medicine, in the country you are working in?
   _______________ (Open question)
3. Please indicate the main policy agencies/institutions that monitor or are involved in overseeing implementation/fostering of Personalised Medicine in the country you are working in. _______________ (Open question)
4. To your knowledge, which are the **research priorities** in the field of Personalised Medicine in the country you are working in? _______________ (open question)
5. Please name important **funding sources** in the field of Personalised Medicine in the country you are working in _______________ (open question)
6. Please name additional **relevant initiatives** (e.g. relevant national or international projects or consortia) related to Personalised Medicine in the country you are working in. _______________ (open question)

**SECTION 3: Facilitators and barriers for collaborations between Europe and China in Personalised Medicine**

1. Are you aware of any collaborations in the field of Personalised Medicine between Europe and China?
   1. Yes*
   2. No
   3. I do not know

*If yes, please indicate:

Name of the project/collaboration __________

Source or website, if available __________

Any additional information you consider useful __________

1. In your view, which are the most relevant **facilitators** **or enabling factors** for EU-China collaborations in

the field of Personalised Medicine? _______________ (open question)

3. In your view, which are the most relevant **barriers** for EU-China collaborations related to Personalised Medicine? _______________ (open question)

4. In your view, please indicate relevant **contextual aspects (social, cultural, economic, ethical, etc.)** to be taken into consideration in EU-China collaborations in the field of Personalised Medicine
 ______________ (open question)

5. In your opinion, which actions should Chinese and European policy makers implement for intensifying EU-China collaboration in the field of Personalised Medicine? __________ (open question)

6. In your opinion, which are the most important priorities and challenge areas towards EU-China collaborations in Personalised Medicine to be considered in the following areas? (Please select up to three areas and specify the respective priorities)

- 1. Citizens’, patients’ awareness and empowerment __________
  2. Health Professionals’ education and curricula __________
  3. Practices and strategies for Personalised Medicine in sustainable health care __________
  4. Big data and ICT Solutions __________
  5. Bringing innovation to market __________
  6. Translating basic to clinical research and Beyond __________
  7. Research Funding __________
  8. Privacy/Ethical regulations __________
